# Supplementary material for: Comparison of phage-derived recombinases for genetic manipulation of Pseudomonas species
Source: Microbiol Spectr. 2023 Oct 26;11(6):e03176-23. doi: 10.1128/spectrum.03176-23 (PMC10714826; doi:10.1128/spectrum.03176-23)
Supplement: Supplemental material — Tables S1 and S2; Fig. S1 to S5. [file spectrum.03176-23-s0001.pdf]

Supplemental Materials:

Comparison of phage-derived recombinases in genetic manipulation of *Pseudomonas* species

Madison J. Kalb, Andrew W. Grenfell, Abhiney Jain, Jane Fenske-Newbart, and Jeffrey A. Gralnick*

*Address correspondence to Jeffrey A. Gralnick, gralnick@umn.edu

Table S1. Rif^R mutations in *Pseudomonas.*

Table S2. Overview of select recombineering efforts to date in *Pseudomonas spp.*

Figure S1. Effect of wash buffer on transformation efficiency.

Figure S2. Structure and location of Rif^R binding pocket.

Figure S3. Growth defect of *rpoB* Q518L mutants in *P. protegens* CHA0.

Figure S4. Effect of phosphorothiolation of oligonucleotide.

Figure S5. Multiple sequence alignment of *Pseudomonas* Ssb amino acid sequences.

Table S1. Rif^R mutations in *Pseudomonas*

Strain	Position	Nt change	Mutation	# mutants
P. protegens Pf5	1561	G → A	D521N	1
P. protegens CHA0	1562	A → G	D521G	1
P. protegens Pf5	1562	A → G	D521G	7
P. putida KT2440	1562	A → G	D521G	10
P. protegens CHA0	1567	A → G	N524D	1
P. protegens CHA0	1580	C → T	S527F	1
P. protegens CHA0	1599	C → T	Silent	1
P. protegens CHA0	1650	C → T	Silent	1
P. protegens Pf5	1706	C → T	P569L	1
P. protegens Pf5	1736	C → T	S579F	1

Table S2. Overview of select recombineering efforts to date in *Pseudomonas spp.*

Strain	System reported with highest efficiency	Substrate	DNA Load† (approx.)	Genetic Target	Efficiency*	Reference
P. aeruginosa PA14	pUCP-RedS (Arabinose-induced λ Red Beta, Exo, Gam)	PCR product with 100 bp homology arms	7×10^{12} copies	Δ*pqsC*::kan (800 bp insertion)	3.4×10^{-9}	(28)
P. syringae pv. tomato DC3000	Constitutively expressed RecT/E from *P. syringae* pv. syringae B728a	ssDNA with 40 bp homology arms	1.2×10^{14} copies	4 bp mutation K43R in *rpsL*	2.4×10^{-4}	(21)
		PCR product with 91 bp homology arms	5×10^{12} copies		4.5×10^{-9}	(21)
P. putida KT2440	M-toluic acid inducible λ Red Beta, Exo, Gam	PCR product with 500 bp homology	5.4×10^{11} copies	Δpp_0589::kan (800 bp insertion)	2.7×10^{-7}	(35)
	M-toluic acid inducible RecT/E from *E. coli* MG1655	PCR product with 100 bp homology	2.6×10^{12} copies	Δpvd::tetA (1.1 kB insertion)	5×10^{-8}	(36)
	M-toluic acid inducible λ Red Beta	ssDNA with 50 bp homology	1.2×10^{14} copies	1 bp mutation (kan knock-in)	2×10^{-3}	(12)
P. putida KT2440	Constitutively expressed λ Red Beta	ssDNA with 45 bp homology	3×10^{14} copies	4 bp Q518L change in *rpoB*	2×10^{-5}	This study
P. putida EM42 (KT2440 derivative)	3MB inducible Ssr T1E_1405 from *P. putida* DOT-T1E	ssDNA with 45-50 bp homology	2.9×10^{13} copies	100 bp deletion in *pyrF*	5×10^{-4}	(15)
P. protegens Pf-5	Rhamnose inducible RecT/E from *P. syringae* pv. syringae B728a and λ Red Gam	PCR product with 100 bp homology	1×10^{13}	Gentamicin cassette onto plasmid	1.5×10^{-6}	(22)
	Constitutively expressed λ Red Beta	ssDNA with 45 bp homology	3×10^{14} copies	15 bp D521P change in *rpoB*	1×10^{-4}	This study

P. protegens CHA0	Constitutively expressed λ Red Beta	ssDNA with 45 bp homology	3×10^{14} copies	15 bp D521P change in *rpoB*	5×10^{-4}	This study

*Efficiency selected from highest reported in single study or calculated based upon data provided.

† Calculated based on reported DNA substrate length and amount using following equation

$$Number\ of\ copies = \frac{Amount\ (ng) \times 6.022\ \times 10^{23}}{Length\ (bp)\ \times 1 \times 10^{9}\ \times Mass\ of\ DNA\ bp}$$

, where mass of DNA bp is estimated as 660 for dsDNA, and 330 for ssDNA when sequence is not provided.

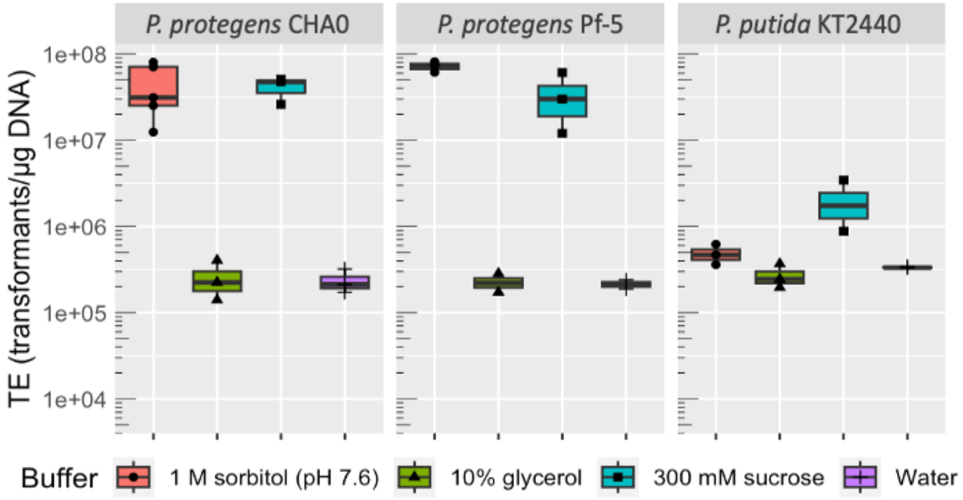

Figure S1. Effect of wash buffer on transformation efficiency. 4 mLs of log-phase cells were harvested via centrifugation and washed 3 times in 1 mL of wash buffer indicated. 250 ng of pBBR1-MCS2 vector purified from GM2163 was introduced into the final cell suspension and electroporated at 12 kV/cm. Cells were recovered in 1 mL of LB for 2 hours and plated on LB plates with or without kanamycin. Transformants were quantified after 2 days of growth.

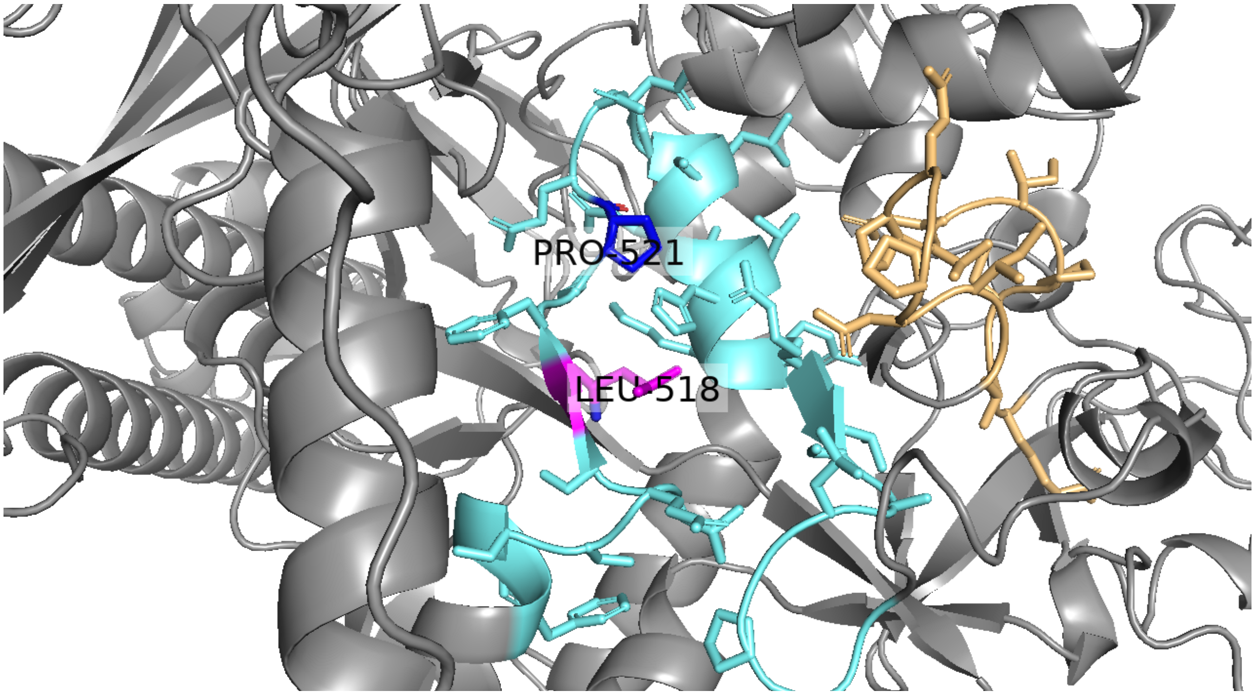

Figure S2. Structure and location of Rif^R binding pocket. Cluster I is indicated by cyan, and Cluster II is indicated by tan. The Q518L mutation (pink) and D521P mutation (navy) were mapped onto the existing structure using the Pymol wizard tool. Structure was generated using *P. putida* ATCC 47054 *rpoB* structure (accession no.: Q88QP2).

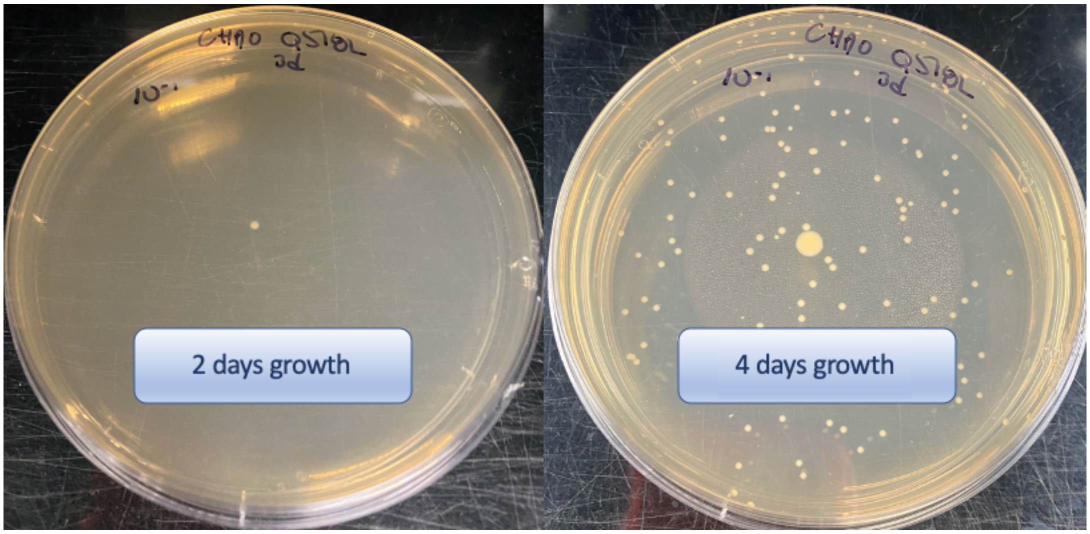

Figure S3. Growth defect of *rpoB* Q518L mutants in *P. protegens* CHA0. The same plate of *P. protegens* CHA0 pictured 2 days (left) and 4 days (right) after recombineering using λ-Red Beta and a recombinogenic oligo encoding a Q518L point mutation in the *rpoB* gene. All delayed growth colonies (n=19) sequenced were found to carry the Q518L point mutation.

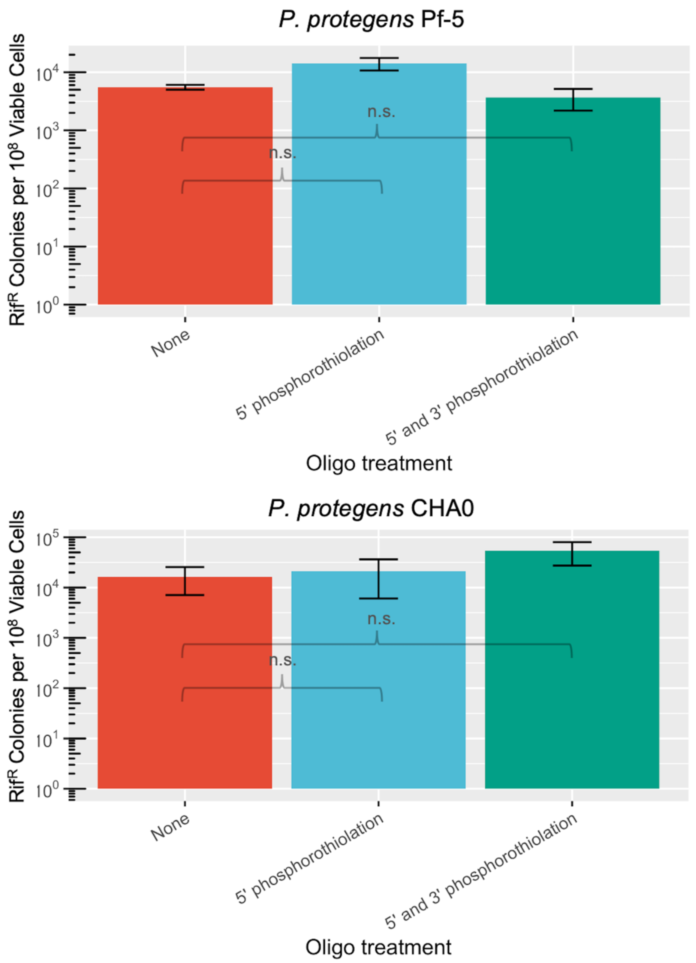

Figure S4. Effect of phosphorothiolation of oligonucleotide. Log phase cultures of *P. protegens* Pf-5 and *P. protegens* CHA0 expressing *E. coli* λ Red Beta were electroporated with 15 µg of oligonucleotide encoding a D521P point mutation without phosphorothioate bonds, with 4 5' end phosphorothioate bonds, or 4 5' and 3' each phosphorothioate bonds, and the cell mixture recovered for 3.5 hours in LB before plating on rifampicin. Rif^R colonies and total viable colonies were counted after 2 days of growth. Significance values are indicated for a Mann-Whitney U test between two groups, where * $p < 0.05$; **$p < 0.01$; ***$p < 0.001$; and; ns, not significant.

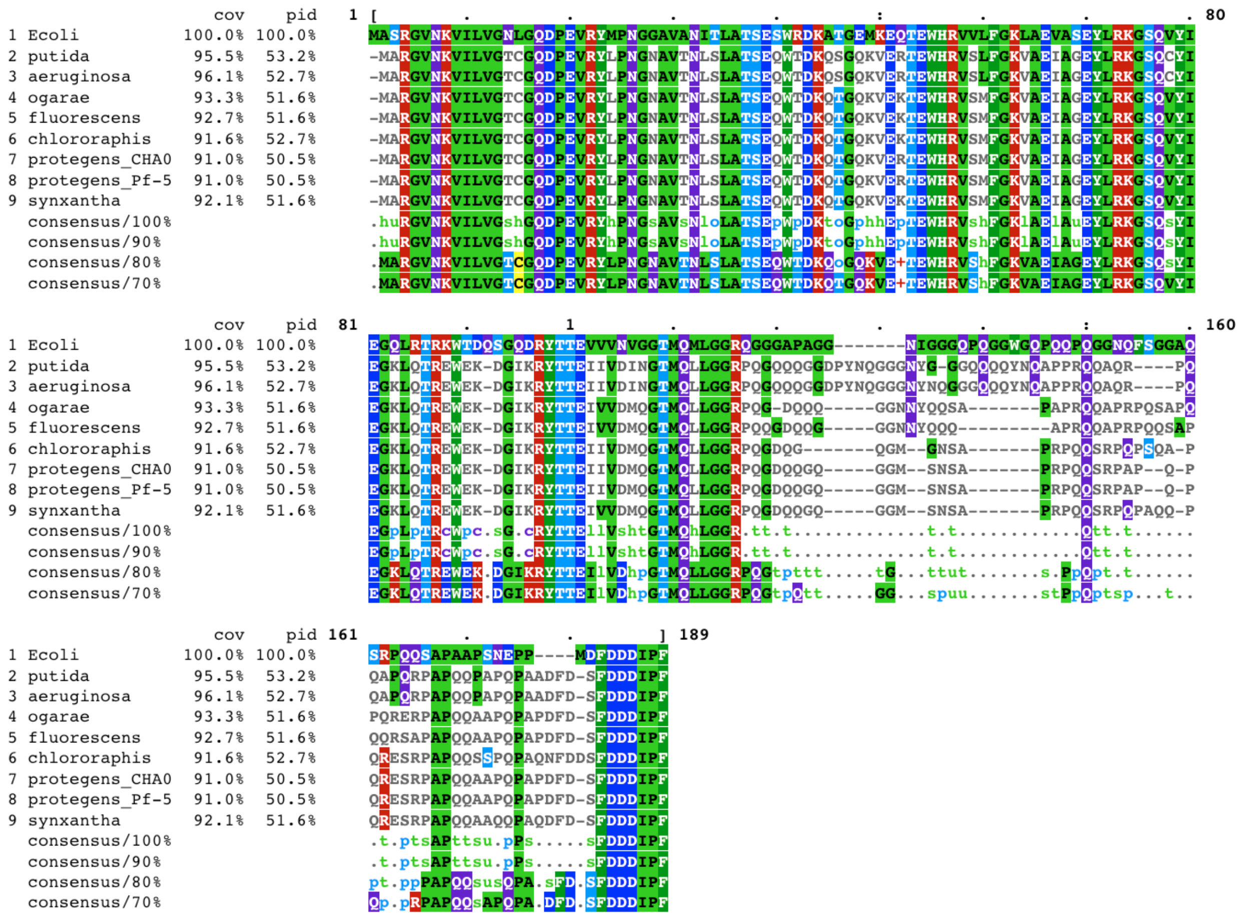

Figure S5. Multiple sequence alignment of *Pseudomonas* Ssb amino acid sequences. MSAs were generated using Clustal Omega from EMBL-EBI using standard parameters.
